# A Systematic Review with Framework Synthesis of the Ways That Urban Environments Influence Opportunities for Healthy and Sustainable Mobility in Older Age

**DOI:** 10.3390/ijerph192013014

**Published:** 2022-10-11

**Authors:** Tracey Ma, Jinhee Kim, Myron Anthony Godinho, Evelyne de Leeuw, Kathleen Clapham, Conrad Kobel, Rebecca Ivers

**Affiliations:** 1 School of Population Health, UNSW Sydney, Sydney, NSW 2052, Australia; 2 Centre for Health Equity Training, Research & Evaluation (CHETRE), UNSW Sydney, Sydney, NSW 2052, Australia; 3 Healthy Urban Environments Collaboratory, Maridulu Budyari Gumal SPHERE, Liverpool, NSW 2170, Australia; 4 Ngarruwan Ngadju First Peoples Health and Wellbeing Research Centre, University of Wollongong, Wollongong, NSW 2522, Australia; 5 Australian Health Services Research Institute (AHSRI), University of Wollongong, Wollongong, NSW 2522, Australia

**Keywords:** healthy ageing, well-being, age-friendly transportation, community mobility, access, healthy city, age-friendly city, age-friendly environment

## Abstract

Supporting older people’s use of sustainable transport is important for both population health and sustainable development, especially in the context of global population ageing. This systematic review identifies individual and environmental factors that influence older people’s sustainable transport use and synthesises findings using a framework approach. Factors influencing older people’s walking (n = 10 studies), bus use (n = 11), community transport use (n = 1), bicycling (n = 1), and e-bicycling (n = 1) were found to be physical, geographical, facility-based, economic, time-based, fear-based, space-based, information-based, or interpersonal. Many factors were common across transport modes. One reason for this is that environmental features designed to facilitate the use of one particular transport mode also influenced the use of other modes (e.g., bus shelters influence not only bus use but also walking as they provide pedestrian seating). Thus, environments need to be considered from the perspective of multiple, different types of road users. Another reason is that many factors related to the ways individuals experienced their environment (e.g., finding information guiding behaviour in public spaces to be unclear), regardless of any specific transport mode. This review highlights the important need for greater cross-sectoral action and input from older people.

## 1. Introduction

Transportation influences health in multiple ways; it impacts people’s access to goods, services, and life opportunities [1], social interactions [2], physical activity levels [3], air pollution exposures [3], and road injury risks [3]. These influences occur across the life course; however, as human populations are ageing [4], there is increasing emphasis on the role of transportation in facilitating older people to live healthy lives [5,6,7]. The World Health Organization (WHO) defines age-friendly transportation in instrumental terms, focusing on older people’s “ability to move about the city” for the purposes of “social and civic participation and access to community and health services” [8]. This definition suggests that, of the various influences of transportation on health, supporting older people’s mobility and enabling their access to activities in society is of primary importance to healthy ageing.

Supporting older people’s mobility through sustainable transport, such as active transport, public transport, and community transport, rather than private motor vehicles is of particular importance to health. Recent modelling studies have found a net health benefit of increased active and public transport use [9,10,11]. While these studies did not focus exclusively on older people, the estimated health benefits for older people are likely to be higher, given older people’s higher health risks and greater likelihood of benefitting from increased physical activity [9]. The use of sustainable transport also enables older people to access resources that support health, such as green space [12], health care [13], and grocery stores [14]. Furthermore, it increases older people’s capacity to carry out everyday activities of value [15], particularly discretionary trips that contribute significantly to quality of life [16]. In addition to the health imperative, supporting older people’s sustainable transport use is congruent with the need to manage the environmental footprint of cities for sustainable global [17] and urban [18] development.

Despite the clear advantages of moving to sustainable transport, in reality, older people rely heavily on private motor vehicles [19,20,21,22] and are not well supported to use other transport modes. For example, the evidence suggests that public transportation is insufficiently equipped to meet the needs of older people [23,24], with the vast majority of transport planning authorities anticipating that older people will be resigned to car dependency as a result of inadequate current plans [24]. Thus, informed efforts to support older people’s sustainable transport use are urgently needed. It is worth noting that different sustainable transport modes have different implications for older people, with, for example, active transport associated with greater physical activity benefits but also greater exposure to road injury risks and public transport enabling greater travel distances but requiring travel to occur at certain times. Despite this, it is important for older people to have opportunities to use these different sustainable transport modes to maintain and support their mobility.

Several systematic reviews have sought to identify environmental factors that influence older people’s sustainable transport use [25,26,27,28,29]. However, existing studies are limited as they tend to overlook individual differences, and, specifically, the interplay between individual factors and environmental factors, in older people’s sustainable transport use [30,31]. As there is more heterogeneity among older people than among young and middle-aged adults due to individually specific, age-related changes in activity patterns, daily routines, and physiological and psychosocial processes [32,33], individual differences are especially important to consider. The limited attention to this means that our knowledge about the influences on older people’s sustainable transport use is incomplete [25,26,27].

The aim of this systematic review is to identify factors that influence older people’s sustainable transport use in a way that is sensitive to both individual and environmental factors. To do so, this review draws on a framework of transport factors that affect people’s access to activities in society [34]. By emphasising factors that affect *access to activities in society*, this framework aligns well with the WHO definition of age-friendly transportation, making it particularly relevant for studying older people’s transport use. By comprising 4 categories of environmental factors and 3 categories of individual factors, this framework is uniquely suited for illuminating the ways that individuals experience their environment to shape their opportunities for sustainable mobility.

## 2. Methods

### 2.1. Identification of Studies

The following databases were searched between 30 July and 1 August 2019 for peer-reviewed articles: Medline (Ovid, New York, NY, USA), Urban Studies Abstracts (EBSCOhost, Ipswich, MA, USA), Scopus, and Web of Science. Collectively, these databases cover the subject areas of public health, social sciences, built environment, and transportation. Keywords corresponding to the older population and the mobility topic were used to conduct the search (see Appendix A). Subject headings were also used when searching Medline. This search strategy was designed with the input of two information specialists, one of whom is part of the authorship team. The search strategy underwent four pilot rounds and prioritised specificity over sensitivity, with one exception: although denoting an aggregate measure, the term ‘walkability’ was included in the search terms because it is sometimes used more loosely than literally. Additional articles were identified from the reference list of included articles and by contacting the authors of included articles for other articles published from the same study.

### 2.2. Selection of Studies

Retrieved citations were uploaded to Covidencefor two-stage screening: first, based on the title and abstract; then, based on the full text. Both stages were conducted by two authors, independent of each other. Any disagreements or queries were solved by a discussion between the two authors.

### 2.3. Inclusion of Studies

This systematic review included peer-reviewed articles published in English from 2009 onwards. The most recent ten-year period was chosen as this limits findings to most recent studies, including those that build on earlier work.

Articles were included if they (1) reported primary research on: the impacts of community- or system-level interventions on the use of sustainable transport among community-dwelling, ambulatory older people (i.e., not person-level interventions like strength training); or the barriers or facilitators to the use of sustainable transport among community-dwelling, ambulatory older people; (2) were conducted in urban or sub-urban areas in Australia, Canada, or the United States, which share similarities in the proportion of population age 65 and over, proportion of population living in urban areas, urban population growth rates, and urban sprawl patterns [35,36]; and (3) provided the level of detail necessary for synthesis into the a priori framework.

In practice, the third criterion excluded studies on interventions that did not identify the component(s) of the intervention (e.g., reduced bus fares as the intervention component needs to be identified as it corresponds to ‘economic factors’ in the framework). It also excluded studies that used aggregate measures (e.g., walkability, connectivity) to describe the environment instead of identifying the specific environmental features of relevance. Aggregate measures may not be relevant for older people because they omit factors important for understanding older people’s engagement with their environment [37,38], such as spatial familiarity and suitability for assistive technologies, and are generally found to be misrepresentative of actual human behaviour [39]. They also cannot capture specific environmental features, such as benches and curb cuts, that are important for older people’s mobility [25]. Even if aggregate measures *are* relevant for understanding older people’s mobility, they are not directly actionable nor modifiable [40].

We assumed the definition of older people used by study authors and included articles of any study design with either objective or subjective measures, as they capture different aspects that are important for understanding older people’s mobility [25,26,41]. We excluded articles that used a spatial unit of analysis with an older person descriptor (e.g., census tracts with a certain proportion of older people). We also excluded articles that conceived of sustainable transport use solely as a means for physical activity.

### 2.4. Appraisal of Studies

The quality of included articles was assessed using the Mixed Methods Appraisal Tool (MMAT) Version 2018 [42]. The MMAT is efficient, reliable, and useful [43,44,45] with good content validity [46]. Appraisal of papers were conducted by two authors, independent of each other. Any disagreements or queries were solved by a discussion between the two authors. All articles were included regardless of quality, as the value of individual articles might only become recognisable at the point of synthesis rather than during appraisal [47].

### 2.5. Analysis of Studies

The following data, where available, were extracted from each article by the first author using NVivo 12 Pro: first author last name, publication year, city, country, objectives, study type (i.e., qualitative, quantitative, mixed methods), study design (e.g., cross-sectional), analytic technique, data measures, data sources, sample size, sampling strategy, transport mode(s) studied, scale of movement (e.g., neighbourhood), purpose of trip (e.g., to the doctor), factors influencing older people’s sustainable transport use, the direction of influence of each factor (i.e., positive, negative), the socio-demographic characteristics of the sample according to Progress Plus [48] and including functional status and access to a private vehicle, and the socio-psychological characteristics of the sample (e.g., familiarity, past experience, habits). It was further noted whether the article was an evaluation study and whether it focused only on older people. The extracted data were verified by another author for 30% of the included articles.

The coding of factors was guided by Hsieh and Shannon’s directed content analysis procedure [49]. A broad interpretation was adopted, thereby including instances where the factor prevented (e.g., lack of footpaths precluded walking) or hindered (e.g., lack of shade cover on direct walking route necessitated using alternative routes) actual travel behaviour, as well as instances where the factor did not affect actual travel behaviour but impacted the travel experience (e.g., steps at bus entry resulted in perceptions of low usability). Findings from quantitative studies that were not statistically significant were not included.

### 2.6. Synthesis of Studies

Data were synthesised using a framework approach, drawing on a framework of transport factors that affect people’s access to activities in society [34]. According to this framework, relevant factors fall within 7 broad categories, 4 of which comprise environmental factors and 3 of which comprise individual factors (see Table 1) [34]. A framework synthesis is a deductive approach that uses an a priori framework to categorise each key factor identified in the source articles [50]. It offers a means to reinforce, critique, and elaborate on an existing framework that may have been conceived for a different but relevant purpose [51]. While a framework synthesis is largely a deductive approach, new categories may be incorporated as they emerge from the data [52]. We applied the ‘best fit’ approach, which differs from other versions of framework synthesis in that the framework was selected due to it being ‘good enough’ or ‘best of what’s available’ rather than it being ‘perfect’ or ‘an exact match’ [51,53]. The intent was to apply an existing framework, rather than to develop a framework.

## 3. Results

### 3.1. Selection of Studies

**Figure 1:**
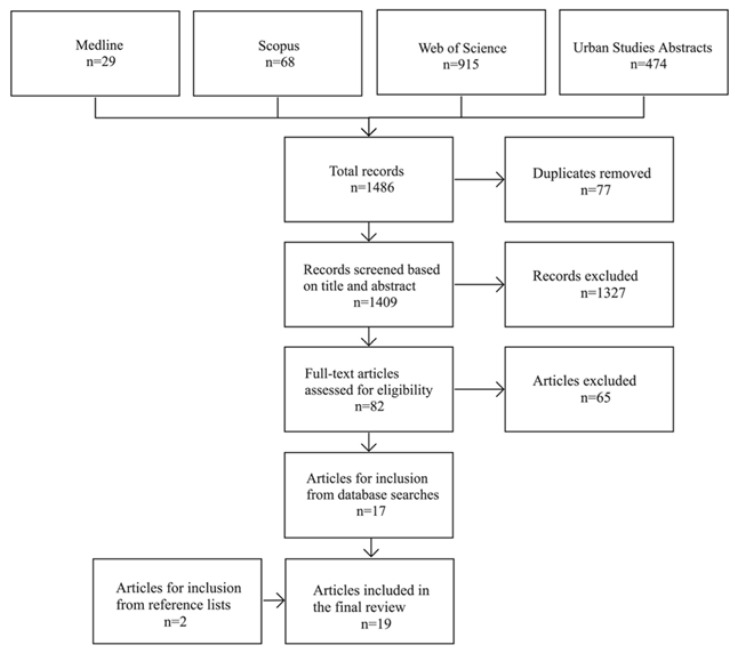
PRISMA flow diagram depicting the article selection process.

The database search returned 1486 records. After removing duplicates and screening for relevance, 82 records were retained for eligibility assessment. Of those, 17 articles met the inclusion criteria. This process is detailed in Figure 1. An additional 2 articles were included after searching the reference lists and contacting the authors. In total, this review included 19 articles comprising 11 studies.

### 3.2. Characteristics of Studies

Of the 19 articles, 9 reported data from Australia [54,55,56,57,58,59,60,61,62], 8 from Canada [63,64,65,66,67,68,69,70], and 2 from the United States [71,72]. The vast majority included only older people in their sample, defined as age 55 or older [59,60,61,62,66,71], age 60 or older [54,57,69,70,71], age 65 or older [58,64,65,67,68], or age 75 or older [63]. However, 2 articles included the general population in their sample but reported separately on older people, defined as age 60 or older [55,56]. Of the 19 articles, 3 used mixed methods [57,67,70], 13 used qualitative methods [56,59,60,61,62,63,64,65,66,68,69,71,72], and 3 used quantitative methods [54,55,58]. Three were evaluation studies [54,55,67]. Walking was referenced in 10 articles [58,60,61,62,63,64,65,68,69,72], bus use in 11 articles [54,55,56,57,59,60,61,62,64,65,67], community transport use in 1 article [71], bicycling in 1 article [70], and e-bicycling in 1 article [66]. Five articles referenced multiple transport modes (despite some of their titles alluding to only one mode) [60,61,62,64,65]. Table 2 presents the characteristics and findings of included articles.

### 3.3. Appraisal of Studies

Individual item scores for each article is provided in Appendix B. Table 3 presents a summary of the quality assessment scores across all articles.

### 3.4. Synthesis of Studies

Appendix C presents the factors that influence older people’s use of each sustainable transport, grouped by category. Several factors were identified that do not fit into the categories of the a priori framework. These factors relate to other road users and to the communication of information and stipulation of rules. We report on these factors under two new categories: “Interpersonal Factors” and “Information-Based Factors”.

The factors with the strongest relevance to walking were space-based factors, followed by physical factors, interpersonal factors, facility-based factors, geographical factors, information-based factors, fear-based factors, and time-based factors. The factors with the strongest relevance to bus use were physical factors and interpersonal factors, followed by information-based factors, economic factors, geographical factors, space-based factors, and time-based factors. The factors affecting community transport use were time-based factors. The factors with the strongest relevance to bicycling were fear-based factors, followed by interpersonal factors and physical factors. Finally, factors with the strongest relevance to e-bicycling were interpersonal factors, followed by information-based factors, physical factors, and fear-based factors.

There are influences on older people’s sustainable transport use that are common across modes. The lack of appropriate road infrastructure, such as dedicated pathways free of obstacles, is a barrier to walking [60,61,62,63,68,72], bicycling [70], and e-bicycling [66]. This is also a barrier to bus use [57], as obstacles affect the accessibility of routes that bus users take to reach bus stops. Likewise, steep topography on the path to bus stops is a barrier to bus use [57,61,62], as it is for walking [60,61,62]. Shade cover and street seating also influence walking [61,62,63,64,68,69,72] and bus use [56,57], although for the latter shade cover and street seating is associated with waiting for the bus and is specific to bus shelters and stops. The presence of bus shelters and stops also facilitate walking as they provide seating options for pedestrians [64]. While the location of destinations is not a factor that influences bus use, unlike for walking [58,62,64,65,68], whether bus routes connect to desired destinations and bus stops are available close to destinations are [56,59,60,61,64]. In some instances, the inability to access desired destinations by walking and bus use resulted in travel to other areas by car [61,62].

Vehicle traffic is a barrier to both walking [64,65,68] and bus use [57], with the latter specific to the route taken to reach bus stops. Likewise, the fear of sharing the road with cars and the fear of motor vehicle collisions are barriers common to bicycling [70] and e-bicycling [66]. Overcrowding by other road users is also a common barrier [57,60,61,62,68,72], making public spaces difficult to navigate and resulting in a fear for personal safety (e.g., getting bumped into) [61,68] and a change in travel behaviour to avoid crowds [61]. The presence of other road users can sometimes be a positive factor and other times a negative one. In the first instance, the presence of other road users signal a potential for social interaction [67,72]. In the second instance, it signals the potential for conflict, for example when there are cyclists, rollerbladers, and skateboarders on shared pathways [62,64,65]. The conduct of other road users is a barrier to walking [68,72], bus use [56,57], bicycling [70], and e-bicycling [66]. However, the helpful behaviour of other bus users or the bus driver is a facilitator to bus use [54,56,57].

Uncertainty about the rules or etiquette governing behaviour is a barrier to walking [62,64] and e-bicycling [66]. Similarly, a specific rule or etiquette (e.g., walking facing traffic and exiting the bus at rear doors) is a barrier to walking [64] and bus use [64] in that it is deemed unsafe and thus older people do not want to abide by it. The timing of service delivery is a barrier to both bus use [54,55,56,57,60,61,62] and community transport use [71]. Finally, a lack of co-ordination between transportation modes, such as buses and trains is a barrier [56]. The use of shuttle buses to enhance connections with major routes, especially during off-peak hours, is a positive factor [56].

## 4. Discussion

### 4.1. Key Findings

Factors that influence older people’s walking, bus use, community transport use, bicycling, and e-bicycling were found to be numerous and fall within the 7 categories of the a priori framework (Table 1) [34]. Two other categories (“Interpersonal Factors” and “Information-Based Factors”) were added to the a priori framework, resulting in an extended framework.

Many factors that influence older people’s sustainable transport use were found to be common across modes. Common physical factors included topography and the availability of appropriate road infrastructure, such as footpaths and bike lanes. Common space-based factors included obstacles on pathways, shade cover, and street seating. Common interpersonal factors included overcrowding, vehicle traffic, the presence of other road users, and the conduct of other road users. Common information-based factors included instances where the information guiding travel behaviour was unclear or perceived to be restrictive. Common time-based factors included service schedules and wait times. Finally, common fear-based factors included the fear of sharing the road with cars and the fear of motor vehicle collisions.

Interestingly, aside from physical factors (i.e., transport system and built environment) and space-based factors (i.e., design, surveillance, and management of public space), most of the factors that are common across modes related to the ways that individuals experience their environment. For example, time-based factors (e.g., infrequent, irregular, or unsuitable service times [56,60,61,62] and unpredictable or excessive waiting times for services [56,61,62,71]) pertain to older people’s lack of control over time when interacting with services in a given place. These factors suggest that there are significant conflicts between scheduling of transport services and times that older people wish to travel. Older people have more discretionary time compared to the target users (working population) of transport systems [73] and they tend to make trips during off-peak periods, when transit services are less frequent [74]. This temporal incongruency is constraining to older people’s mobility.

Information-based factors are another example of factors that are common across modes and that related to the ways that individuals experience their environment. In such instances, the rules, norms, and information guiding behaviour in a given place are interpreted or experienced by older people as unclear or inappropriate. These include unclear information at a specific point in a transportation journey, such as bus signage, timetable, and route information [56,57,59,61,62]; unclear information about how a transportation mode is “governed”, such as the rules to follow as a pedestrian [62,64] and how, when, and where to use e-bikes [66]; and the perception that existing requirements or norms are restrictive, such as walking facing traffic which may result in unnecessary exposure to footpath hazards [64] and exiting the bus at rear doors which is deemed unsafe due to being out of the driver’s line of sight [64]. When the rules, norms, and information guiding behaviour are perceived as unclear or inappropriate, the transportation option that they relate to is, subsequently, interpreted to be of low usability and high complexity [75]. Perceptions of usability and complexity are especially salient for older people in unfamiliar environments, and feelings of discomfort or uncertainty may lead them to a retreat into familiar spaces, limiting their mobility [76].

This review also found that environmental features designed to facilitate the use a particular transport mode also influence the use of other modes. For example, the low quality of footpaths (i.e., topography, availability, and freedom from obstacles) is a barrier not only to walking, but also to bus use among older people, as it affects the extent to which bus users are able to reach the bus stop from residences or destinations [57]. Likewise, the presence of bus shelters and bus stops also facilitate walking among older people as they provide seating options for pedestrians [64]. This finding emphasises the importance of considering how environmental features influence the perceptions and behaviours of different types of road users and the extent to which different road users are accommodated in a shared environment. While there are multiple approaches that encourage a more inclusive (of different road users) view of streetscapes (e.g., shared space, movement and place, complete streets), they are not put into practice everywhere. Furthermore, some research suggests that existing urban design approaches do not go far enough to consider the experiences of certain types of road users [77]. At least for walking and cycling, improving the experience of doing so is essential for promoting their uptake [78].

The few findings on multi-modal trips suggest that the extent of service co-ordination between different transport modes (e.g., buses and trains) influence older people’s sustainable transport use. While multi-modal integration may enhance transport service provision for older people, they also introduce greater scope for interruption, delay, or cancellation, making them less predictable and user-friendly [79]. Furthermore, for multi-modal transportation to benefit older people, they must meet additional requirements that do not, for example, demand users to transfer in isolated locations or juggle various sources of information [79,80]. Given that multi-modal transportation requires its users to be more tolerant of complexity, they may not be suited for all older people. Indeed, older people who use multi-modal transportation tend to be active with average or above-average socio-economic and health resources [81]. Thus, while there is value in investing in multi-modal transportation to support older people’s mobility, this investment must not come at the cost of other solutions, such as increasing mixed land use so that older people live in close proximity to destinations and do not need to undertake multi-modal travel. 

### 4.2. Methodological Considerations

A benefit of using a framework to understand the myriad influences on older people’s sustainable transport use is that the framework places no limits on the number of factors nor on their specific nature. Any empirically observed factor is relevant to our understanding as long it can be grouped under a broader category of the framework. In this process, numerous factors can be aggregated into their shared category and new categories can be derived from individual factors [52]. This enables a highly structured and transparent approach to organising and analysing large amounts of data [50,52]. Because the intention is to apply an existing framework to the data rather than to develop a new framework from the data for application elsewhere, concerns about the restricted geographic scope and the limited number of studies might be attenuated. Regardless, the scope of this review may limit the applicability of findings to urban and sub-urban environments within contexts similar to Australia, Canada, and the United States.

By applying a framework that is sensitive to both individual and environmental factors, this review uncovers the ways that individuals experience their environment to shape their opportunities for sustainable mobility. This process provides insight into how individual differences come to matter. While the call for more investigation into individual factors might be interpreted as a call for subgroup analyses using demographic markers such as gender and ethnicity, such analyses would not reveal *how* influences on older people’s sustainable transport use differ between groups of older people, just that it does according to a specified demographic marker. In contrast, this review explores the possibility that individuals with certain observable characteristics, such as being retired and thus having a different ‘time budget’, might find certain factors to be enabling or constraining to their sustainable transport use because of the way they experience their environment. This possibility has been explored to an extent by individual studies (e.g., investigating differences in cycling uptake not by contrasting behaviour between demographic groups but by examining social practices across various identities [82]), but has not been synthesised across multiple studies in relation to older people specifically. However, the limited number of studies included in this review suggests that this possibility, as it pertains to older people, has not been sufficiently explored by primary studies in the first instance. While the synthesis framework does not explicitly account for other factors such as socio-economic status and functional status, such information was sought in the data extraction process (under the categories of “socio-demographic” and “socio-psychological” characteristics). However, this information tended to be reported only as aggregate sample descriptives without consideration of their relevance to individuals’ use of sustainable transport. The limited information on functional status in the primary studies is particularly noteworthy as individual differences in functional status influence mobility both directly and indirectly, with the latter via experiences of the environment.

Regarding the modes of transportation, this review found more studies on, and therefore more factors relevant to, walking and bus use. While neither the search terms nor the eligibility criteria specify the mode of transportation, the words “transit” and “walk” do appear in the search terms. This may have emphasised those modes of transportation, but it should not have limited or excluded others. As such, the fact that most studies focused on walking and bus use is more so a reflection of the frequency with which it was investigated by the primary studies. Similarly, while data on multi-modal trips were extracted where relevant, there were relatively fewer findings on multi-modal trips than there were on individual modes of transportation. As such, data on multi-modal trips were not presented as figures.

### 4.3. Future Research Directions

By applying a synthesis framework sensitive to both individual and environmental factors to existing studies, this research has explicitly attempted to tease out how individual differences affect older people’s sustainable transport use. However, the limited number of studies included in this review confirm the peripherality of such questions in primary studies and suggest a need for further research on how older people experience their environment to shape their opportunities for sustainable mobility. Future research should also consider the role of socio-demographic and socio-psychological factors, with the former affecting an individual’s situation and the latter affecting how an individual acts upon his/her situation and options [83]. While such information may not lead directly to prescriptions for action, they are relevant for framing the policy agenda and the “solutions space” [84]. For example, already, it is clear that older people’s experiences need to be taken into account in efforts to promote sustainable transport use, as, without doing so, the economic, time-based, fear-based, information-based, and interpersonal factors that are specific to individual circumstances and that are equally important to environmental factors will be overlooked, resulting in incomplete efforts to support older people’s sustainable transport use. Some studies were not included in this review as they did not provide the level of detail necessary for synthesis into the framework (e.g., interventions were not reported in a way that identifies the component(s) of the intervention, which would have been a positive factor). Thus, future research should also prioritise reporting quality. Lastly, further research should be undertaken to investigate individual and environmental factors influencing older people’s uptake of multi-modal trips.

## 5. Conclusions

While private motor vehicles play an important role in supporting older people’s mobility, it is crucial that older people have opportunities to use other transport modes. This review identified factors that influence older people’s sustainable transport use common across the individual modes of walking, bus use, community transport use, bicycling, and e-bicycling. This review also encourages a reconceptualisation of features that are designed to support the use of one particular transport mode (e.g., bus stops to facilitate bus use) so that they are considered from the perspective of multiple, different types of road users.

As factors that influence older people’s sustainable transport use extend across multiple domains, there is an opportunity for cross-sectoral actions to support the mobility of older people. These include actions pertaining to the scheduling of or operating hours for medical appointments, which may allow for better congruence with transport services; or to the timing of employment hours, with flex or staggered hours potentially reducing overcrowding on buses or traffic volumes on roads.

This review challenges the assumption of environmental determinism, where the role of the individual is ignored [85], and, instead, pays equal attention to the influences on older people’s sustainable transport use that are borne from the way individuals experience their environment. Just as policy makers and practitioners may be subject matter experts in the land use, built environment, and transportation sectors, older people themselves are subject matter experts in their own experiences of the environment. Thus, there is value in including older people’s voices as inputs into decisions about the provision, location, distribution, and use of public space, infrastructure, and services relevant to sustainable transport use.

## Figures and Tables

**Table 1:** Definition of the categories of factors from the a priori framework.

Category	Definition
*Environmental*	
Physical	Physical barriers associated with the transport system and built environment
Geographical	Peripherality or spatial isolation
Facility-Based	The location of facilities
Space-Based	The design, surveillance, and management of public space
*Individual*	
Economic	Income constraints and inability to absorb travel costs
Time-Based	Time constraints
Fear-Based	Attitudes toward public space

**Table 2:** Characteristics and findings of included articles.

Study ID	Article ID	Reference	Aim	Description	Positive (+) or Negative (−) Influences on Older People’s Opportunities for Mobility
1	1a	Broome et al., 2013 [54]	To evaluate the impact of implementing age-friendly guidelines for public buses on bus use, useability, and social participation for older people. [bus]	Quantitative: The authors analysed self-reported information on demographic characteristics, driving behaviour, bus use behaviour, and bus satisfaction gathered from surveys with non-matched bus users before and after the intervention. They also analysed self-reported information on frequency of bus use, perceived ease of bus use, and social participation gathered from surveys with the same cohort of older people before and after the intervention. Participants were aged 60 or older and lived either in Hervey Bay where the intervention took place or Brisbane, which served as a control site. [Australia]	(+) low floors; flexible route service; helpful bus drivers; frequent bus services;
1	1b	Broome et al., 2012 [55]	To investigate whether the replacement of a fixed route bus service with a flexible route bus service improves the use of, and satisfaction with, buses particularly among older people. [bus]	Quantitative: Using a pre- and post- test design without a control, the authors evaluated a service in Hervey Bay that allowed the bus to be dynamically redirected to go past users’ residences, and which was accompanied by improvements to scheduling. Data on bus use were sourced from ticket sales collected from electronic ticketing records, with older adults identified via the Pensioner ticket type; and data on bus usability were collected from on-board satisfaction surveys, with older adults identified by being aged 60 and over. [Australia]	(+) flexible route service; expanded service hours
1	1c	Broome et al., 2010 [56]	To compare barriers to and facilitators of use of public transport for older and younger adults to determine whether age-friendly guidelines are needed for public transport. [bus]	Qualitative: Using qualitative content analysis, the authors reported on the barriers and facilitators generated and ranked via nominal group technique administered within focus groups of 301 bus and non-bus users, of which 76.7% were aged 60 and older. Focus groups were separated by age. All participants lived in Hervey Bay or Brisbane. [Australia]	(+) helpful bus drivers; frequent services; bus stops close to home and destinations; low floors enabling ease of entry and exit; low cost; timetables and routes easy to understand; bus shelters available to provide shade and seating; bus routes were appropriate with good connections to destinations; simple and flexible ticketing system; co-ordination of services and ticketing with trains, airlines, and other buses; use of mini-buses to connect with major routes or at night (−) unsuitable service times; infrequent/irregular services; lack of connection of routes to desired destinations; bus stops far from home, destination, and each other; presence of steps making entry and exit difficult; driver not parking close to curb making entry and exit difficult; indirect bus routes; excessive wait times between connections; lack of co-ordination with other bus, train and ferry services; needing to change buses; unhelpful bus drivers; lack of bus shelters to provide shade and seating
1	1d	Broome et al., 2010 [57]	To explore the barriers and facilitators to all stages of bus use for older people and their relative impact on bus usability. [bus]	Mixed Methods: The authors reported on the barriers and facilitators identified via nominal group technique administered within focus groups of 227 participants and focused ethnography with 40 participants which involved a pre-trip interview, observation of actual bus use, and a stimulated recall interview. All participants were aged 60 or older living in Hervey Bay or Brisbane. Analyses used a thematic approach. [Australia]	(+) wide bus aisles; helpful behaviour of other road users including helping the participant at risk of falling, stopping a participant’s stepping out in front of a moving car; calling out to the bus driver to avoid overshooting the bus during a bus journey, helping participant who was unable to understand a map (−) steep topography, lack of street crossings, and no or obstructed footpaths on the path to bus stops; absent or poorly designed bus shelters; bus entry and exit made difficult by steps or obstacles on footpaths; narrow bus aisles; mobility device not fitting in bus aisles; inaccessible buttons; no handles or railings; uncomfortable seats; trees for shade; unclean bus shelters; bus stops located far from home; poor visibility of buses; no parking near bus stops; indirect or complicated bus routes; bus routes with poor connections; unhelpful bus drivers; inconsiderate behaviour of other bus users; overcrowding; vehicle traffic between home and bus stop; timetable and route information unclear; no signage; service changes that were unexpected or not communicated; infrequent or irregular service schedules; unsuitable service times; expensive ticketing; payment and purchasing method was difficult
2	2	Nathan et al., 2012 [58]	To examine associations between access to and mix of commercial destinations within the neighbourhood and walking in a sample of older adults. [walking]	Quantitative: The authors modelled the relationship between activity levels as measured via the Active Australia Survey within the state-wide Health and Wellbeing Surveillance System and access to each commercial destination type (food retail, general retail, medical care services, financial services, general services, and social infrastructure) within 400 m and 800 m from participants’ home address as computed using spatial data among 2918 residents of the Perth metropolitan region who were aged 65–84 years. [Australia]	(+) destinations close by
3	3a	Zeitler et al., 2015 [59]	To investigate the use of transportation by older people and its implications for their out-of-home activities within suburban environments. [bus]	Qualitative: The authors analysed mobility patterns gathered from time-use diaries and GPS mapping as well as perspectives and experiences on safety, affordability, availability, accessibility, and walkability gathered from interviews of 13 participants aged 55 or older living in Brisbane. [Australia]	(−) bus stop locations far from intended destination; unaware of public transport service options
3	3b	Zeitler et al., 2012 [60]	To explore if and how suburban environments impact older people’s mobility and their use of different modes of transport. [walking] [bus]	Qualitative: The authors analysed mobility patterns gathered from time-use diaries, GPS mapping, and interviews of 13 participants aged 55 or older living in low density areas in Brisbane. [Australia]	Walk (−) limited footpaths or not having footpaths on both sides of the road; pedestrian crossing time being too fast; uneven footpath surfaces; steep topography Bus (+) bus stop location close to home; frequent services (−) bus stop location far from home; bus stop far from destination; bus being overcrowded; infrequent services
3	3c	Vine et al., 2012 [61]	To explore the effect of the neighbourhood environment and its influence on liveability for older urban residents. [walking] [bus]	Qualitative: The authors analysed mobility patterns gathered from time-use diaries, GPS mapping, and interviews of 12 participants aged 55 or older living in high density areas in Brisbane. Analyses used a thematic approach. [Australia]	Walk (−) uneven footpath surfaces; steep topography; overcrowding; footpaths too close to busy roads; pedestrian crossing signals not long enough; lack of shade; lack of street seating Bus (−) bus stops far from desired destinations; infrequent/irregular services; steep topography when travelling to bus stops; excessive wait times; timetable and route information unclear In General (−) minimal amenity choice within reasonable distance
3	3d	Vine et al., 2012 [62]	To explore neighbourhood walkability as older adults experience this phenomenon through time and space. [walking] [bus]	Qualitative: The authors analysed mobility patterns gathered from time-use diaries, GPS mapping, and interviews of 12 participants aged 55 or older living in high density areas in Brisbane. Analyses were guided by an a priori framework on the built environment’s influence on physical activity. [Australia]	Walk (+) destinations close to home (−) steep topography; presence of cyclists on shared paths and potential for conflict; insufficient time for older people to cross the road; confusion about right of way protocol; narrow footpaths; proximity of footpaths to busy roads and merging of footpaths with vehicle traffic; overcrowding; insufficient green space; insufficient street lighting; lack of shade; lack of street seating; unavailability of clean and safe public toilets Bus (−) indirect routes to get to destinations; lack of awareness about timetable and route information; infrequent/irregular services; steep topography on path to bus stops; excessive wait times; timetable and route information unclear In General (−) minimal amenity choice within reasonable distance
4	4	Gardner, 2014 [63]	To understand how neighbourhoods—as physical and social environments—influence community mobility of older adults. [walking]	Qualitative: The author completed an ethnographic study of 6 participants age 75 or older living in Toronto to understand how neighbourhoods were experienced and what challenges were encountered in negotiating the physical environment. Data were collected over 8 months in auditory, textual, and visual formats on go-along interviews. [Canada]	(−) curbs that were too high or were not properly; footpaths that were not cleared of snow, ice, leaves or branches; uneven footpath surfaces; the lack of street seating; graffiti and debris
5	5a	Grant et al., 2010 [64]	To develop a more thorough understanding of older people’s neighbourhood walking experiences with an emphasis on daily life. [walking] [bus]	Qualitative: The authors thematically analysed the everyday walking experiences as derived from focus groups and interviews of 75 participants aged 65 or older living in Ottawa. [Canada]	Walk (+) bus stops with benches for resting (−) destinations separated by main arterial roads; obstacles such as newspaper boxes and vending displays on footpaths; uncertainty about the rules of the road and needing a “rule book for walking”; the need to walk on the street facing traffic as required by law resulted in exposures to hazards; location of destinations beyond walking distance; inadequate crossing signal times; traffic exhaust; long crossing distances across multiple lanes; vehicle traffic; cyclists, rollerbladers, and skateboarders on footpaths Bus (−) the need to exit at rear of bus; obstacles on footpaths such as snowbanks which made it difficult to enter and exit buses; bus stops located far from destinations
5	5b	Grant et al., 2010 [65]	To examine how urban form and neighbourhood SES inter-relate to affect the experiences of older people who walk in their neighbourhoods. [walking] [bus]	Qualitative: In this comparative case study of 4 neighbourhoods, the authors collected information on where people walked and why, what the supportive and unsupportive aspects of the neighbourhood environment were, and what positive and negative neighbourhood changes affected walking. This information was collected via interviews and focus groups with 75 participants aged 65 or older living in Ottawa. Analyses used a thematic approach. [Canada]	Walk (+) presence of footpaths which separated vehicle and pedestrian traffic, allowed for spontaneous meeting opportunities, and legitimize walking as a form of transportation; presence of recreational pathways; desired destinations were located close by (−) footpath grading too high; hazards on footpaths such as carts and fallen fruit; distance to recreational pathways was too far; lack of grocery store nearby or loss of commercial destinations nearby; the need to cross main traffic roadways to reach destinations; heavy vehicle traffic; cyclists and skateboarders on shared paths Bus (−) obstacles between footpaths and bus entrances/exits; indirect or complicated bus routes
6	6	Leger et al., 2019 [66]	To explore the perceived viability of older adult e-bike adoption in the Canadian context with a focus on the determinants of older adult mobility. [e-bicycling]	Qualitative: The authors conducted interviews with 17 governance and community stakeholders as well as focus groups with 37 participants aged 55 and older with a range of bicycling experience living in Waterloo. Analyses were guided by a theoretical framework that incorporates various life-space locations. [Canada]	(−) poor bicycling infrastructure; fear of motor vehicle collision; perceived stigmatization of older cyclists; concern that other road users may misjudge the speed of e-bikes; belief that cyclists are not treated as respected road users; unfamiliarity with the e-bike technology; confusion about rules of use
7	7	Mah et al., 2017 [67]	To examine the potential effect of a free bus program on travel behaviour of older adults in a suburban municipality. [bus]	Mixed Methods: The authors modelled the results of a questionnaire answered by 131 participants aged 65 or older living in Oakville to determine the socioeconomic, access, and travel behaviour predictors of being impacted by the free bus program. They also thematically analysed older adults’ experiences with the free bus program gathered via interviews with 16 participants aged 65 or older living in Oakville. [Canada]	(+) no cost bus service; helpful bus drivers; social interaction opportunities with other bus users (−) had a cheaper alternative
8	8	Mitra et al., 2015 [68]	To explore the relationship between the neighbourhood built environment and walking among a small group of older adults in a large suburban municipality in Canada. [walking]	Qualitative: The authors thematically analysed the perceptions and experiences of walking of 14 participants aged 65 or older living in Mississauga using data from photographs of barriers or facilitators in the built environment that affected walking, drawings of walking routes, and interviews. [Canada]	(+) presence of footpaths; gradual curb cuts; presence of street seating; shade cover; neighbourhood watch signs; security cameras; presence of recreational pathways; street lights (−) timing of traffic lights and pedestrian signals not congruent with older adults’ walking speed; lack of connectivity to destinations due to dead end streets and cul-de-sacs; limited or unavailable footpaths on both sides of the street; uneven footpath surfaces; obstructions on footpaths like loose stones; busy places or crowds; lack of proximity to destinations; vehicular traffic; concern that drivers do not accommodate pedestrians
9	9a	Ottoni et al., 2016 [69]	To explore how a specific micro-scale feature of the built environment influence older adults’ experiences of mobility and well-being, from the perspective of older adults, and how these experiences both affect and are affected by the social environment of their neighbourhood. [walking]	Qualitative: The authors analysed observational field notes and transcripts from sit-down and walk-along interviews with 28 participants aged 60 or older living in Vancouver. Data were collected on participants’ health, physical activity, travel behaviours, perceptions of their everyday experiences of their social and physical environments, their practices of engaging with their environment, and their local built and social environments. Analyses were guided by an a priori framework on the topics of built environment and mobility. [Canada]	(+) presence of street seating
9	9b	Winters et al., 2015 [70]	To describe the bicycling behaviours of older adults and identify factors that facilitate or deter older adults from bicycling in an ideal environment. [bicycling]	Mixed Methods: From a sample of 193 older adults who were aged 60 or over living adjacent to a planned greenway in downtown Vancouver, the authors quantitatively described bicycling behaviour; determined relationships between bicycling behaviour and demographic, health, physical activity, and social connection status; and compared perceptions of bicycling between those who cycled and those who did not. Data were reported via questionnaire and travel diary. Among a subset of 27 participants, the authors further analysed information on participants’ health, physical activity, travel behaviours, and perceptions of their local built and social environments reported via interview. Analyses were guided by an a priori framework on the topics of built environment and mobility. [Canada]	(+) presence of bike lanes (−) fear of sharing the road with cars; concerns that other cyclists and pedestrians disregard the rules of the road; fear of bike theft
10	10	Adorno et al., 2018 [71]	To examine older adults’ experiences and perspectives regarding transportation mobility. [community transport]	Qualitative: The authors conducted interviews with 15 participants and focus groups with 45 participants on what it means to ‘age well’ in the community. All participants were aged 55 and older living in Arlington. Analyses were guided by an a priori framework based on the World Health Organization’s Age-Friendly Checklist. [USA]	(−) unpredictable wait times for pick up and drop off; requirement to book service in advance; unavailable time slots; restricted service hours
11	11	Gallagher et al., 2010 [72]	To identify the salient factors of the neighbourhood environment that encourage or discourage walking in older, urban African Americans. [walking]	Qualitative: Explored the perceptions of that which “encouraged or discouraged neighbourhood walking” among 21 African American participants aged 60 or older living in Detroit through photovoice-based focus groups. Content and thematic analyses were used. [USA]	(+) presence of familiar and friendly faces; peaceful surroundings; buildings or statues with personal or historical meaning; green space; shade; presence of a senior patrol, police, or security; shovelled footpaths free of obstacles; presence of recreational pathways(−) overcrowding; individuals asking for money; people fighting; vacant houses; overgrown lots; trash; inadequate lighting; fallen trees or branches; criminal activity; footpaths that are abruptly terminated; footpaths overgrown with weeds or icy sidewalks; fear of loose dogs; trails that are isolated or are in poor visibility areas

**Table 3:** Summary of article quality assessment.

Quality Assessment Item	N, Articles for Which Item Applies	n, Articles Scoring ‘Yes’
Category 1: Qualitative studies		
1.1. Is the qualitative approach appropriate to answer the research question?	13	13
1.2. Are the qualitative data collection methods adequate to address the research question?	13	13
1.3. Are the findings adequately derived from the data?	13	13
1.4. Is the interpretation of results sufficiently substantiated by data?	13	13
1.5. Is there coherence between qualitative data sources, collection, analysis and interpretation?	13	10
Category 3: Quantitative non-randomized studies		
3.1. Are the participants representative of the target population?	3	2
3.2. Are measurements appropriate regarding both the outcome and exposure/intervention?	3	2
3.3. Are there complete outcome data?	3	0
3.4. Are the confounders accounted for in the design and analysis?	3	0
3.5. During the study period, is the intervention/exposure administered as intended?	3	0
Category 5: Mixed methods studies		
5.1. Is there an adequate rationale for using a mixed methods design to address the research question?	3	1
5.2. Are the different components of the study effectively integrated to answer the research question?	3	1
5.3. Are the results adequately brought together into overall interpretations?	3	1
5.4. Are divergences and inconsistencies between quantitative and qualitative results adequately addressed?	3	1
5.5. Do the different components of the study adhere to the quality criteria of each tradition of the methods involved? *	3	1

* Each of the 3 mixed methods articles were also assessed according to the quality criteria for their respective qualitative and quantitative components. The summary of their individual components is not presented here.

**Figure A1:**
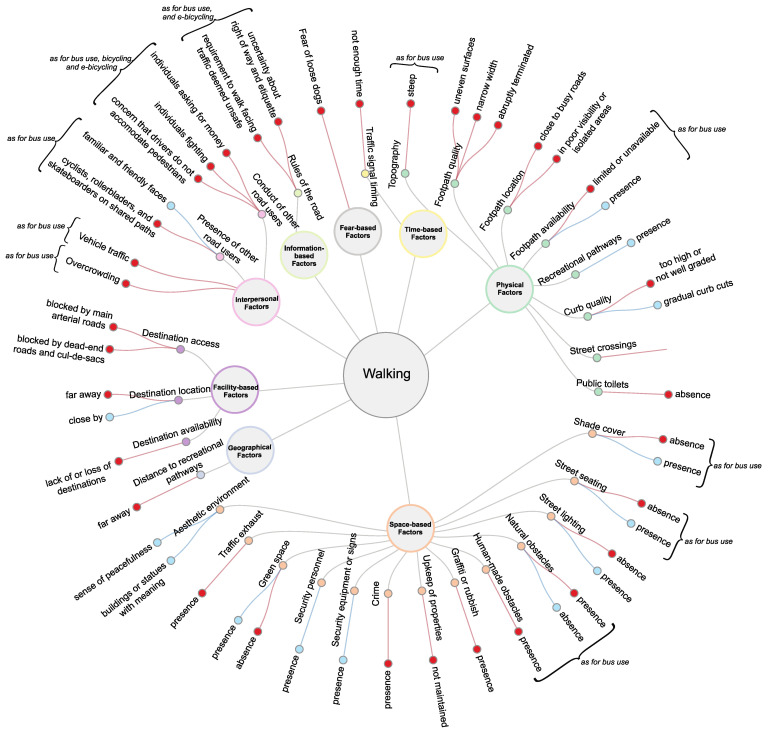
Radial dendrogram depicting the influences on older people’s walking, as derived from 10 articles.

**Figure A2:**
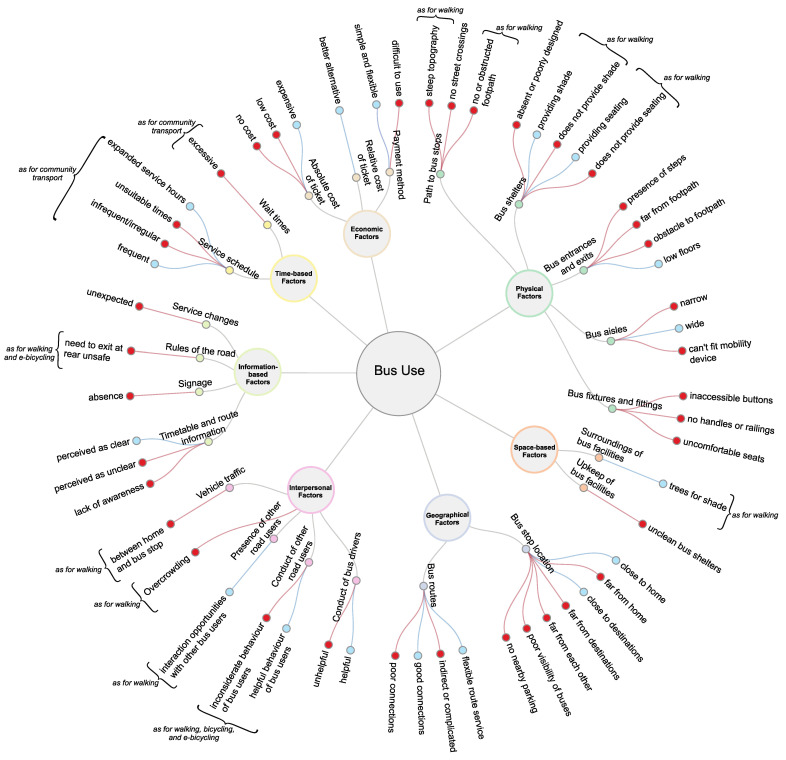
Radial dendrogram depicting the influences on older people’s bus use, as derived from 11 articles.

**Figure A3:**
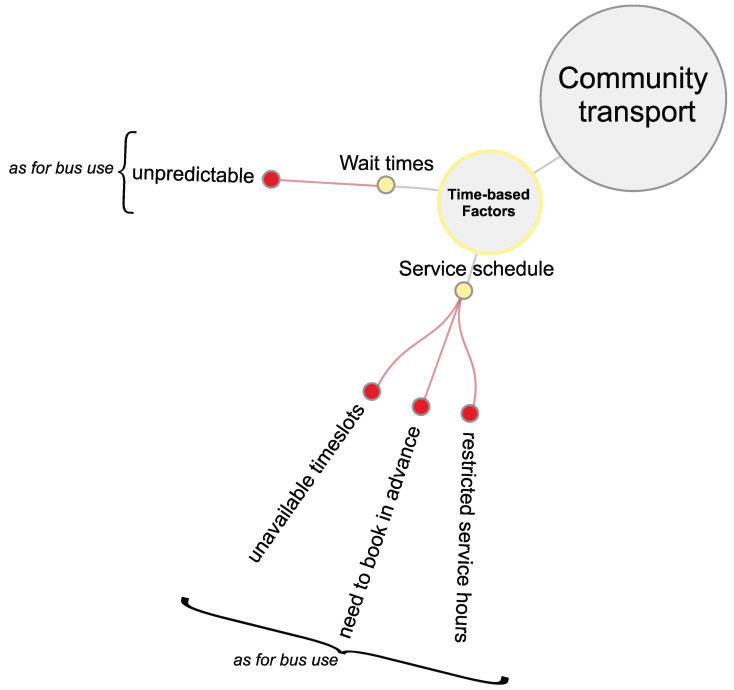
Radial dendrogram depicting the influences on older people’s community transport use, as derived from 1 article.

**Figure A4:**
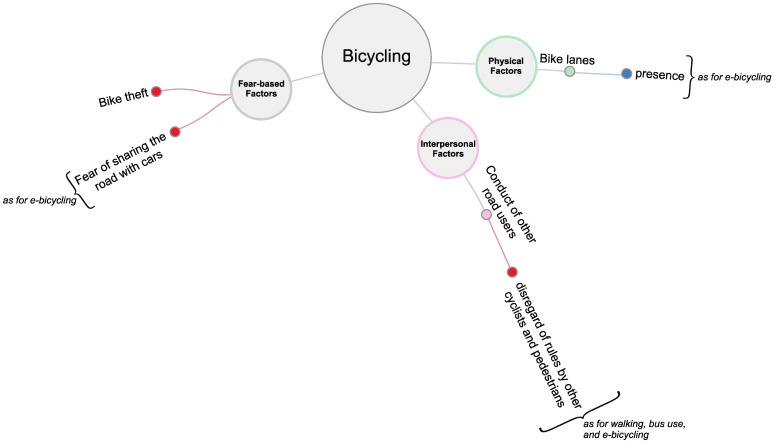
Radial dendrogram depicting the influences on older people’s bicycling, as derived from 1 article.

**Figure A5:**
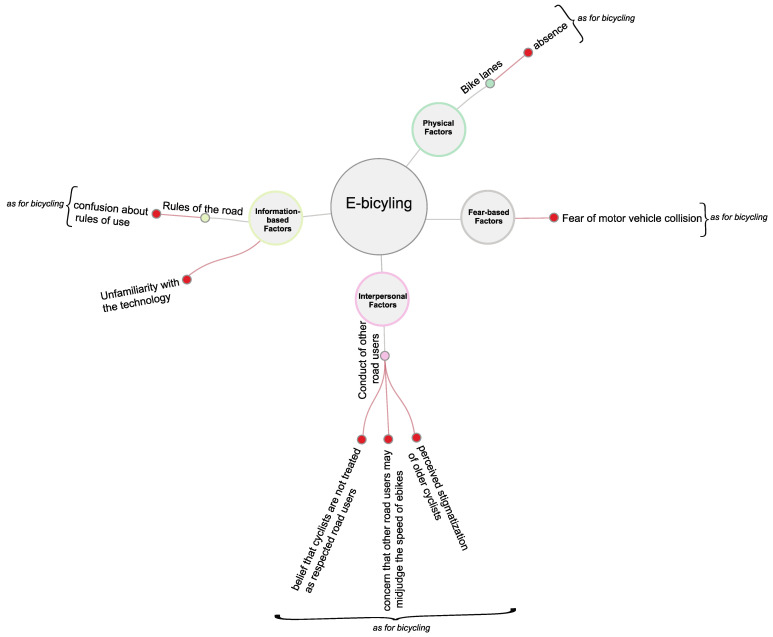
Radial dendrogram depicting the influences on older people’s e-bicycling, as derived from 1 article.

## Data Availability

Not applicable.

## Appendix A

**Table A1:** Search Terms.

#1	Population	elderly or seniors or “older adults” or “older people” or “older persons” or “older citizens” or “older residents” or “older age” or “late life” or “later life” or ag*ing
#2	Concept	“urban accessibility” or “community accessibility” or “transport accessibility” or “transit accessibility” or “urban connectivity” or “transport connectivity” or “transit connectivity” or walkability or “urban mobility” or “community mobility” or “life space mobility” or “life-space mobility” or “transport mobility” or “transit mobility” or (transport OR transportation OR transit OR mobility) W/5 (inclusion OR exclusion OR equity OR inequity OR equitable OR inequitable OR justice OR injustice OR disparity OR polari?ation OR disadvantage) or “equitable use” or “universal design” or age-friend* or “age friendly” or “age friendliness”
#3	Exclusions	“air transport” or “air transportation” or “air travel” or “residential mobility”
*#1 (AB, KW) **AND** #2 (AB, KW) **NOT** #3 (AB, KW)* *Limit to: articles; English; USA, Canada, and Australia; 2009*		

## Appendix B

**Table A2:** Quality of Included Papers.

**Broome et al., 2013** [54] **[Study ID: 1; Paper ID: 1a]**				
Quantitative Non-Randomized	Yes	No	Can’t Tell	Comments
3.1. Are the participants representative of the target population?			X	While older non-bus users are specified to be within the target population, it is not clear how they were sampled. Study 1 utilised on board surveys, which is limited to bus users. Study 2 might have sampled non-bus users as the cohort came from another study, however, this was not specified. There was no information on attempts to achieve a sample of participants that represents the target population.
3.2. Are measurements appropriate regarding both the outcome and exposure/intervention?	X			
3.3. Are there complete outcome data?			X	
3.4. Are the confounders accounted for in the design and analysis?			X	In Study 2, participants were asked to rate whether they were using the buses more, the same, or less frequently compared with 3 years ago. Changes in this measure could be a result of other factors, not necessarily attributable to the intervention, such as changes in the need for bus services. However, these other factors were not asked about in the data collection, and therefore could not be accounted for in the analysis. The explanation of what was modelled is also unclear. A regression model was employed to address outcomes 1 and 3 about satisfaction and frequency of use, yet the authors explained the outcome to be location and time period whereas satisfaction and frequency of use were the explanatory variables.
3.5. During the study period, is the intervention/exposure administered as intended?			X	
**Broome et al., 2012** [55] **[Study ID: 1; Paper ID: 1b]**				
Quantitative Non-Randomized	Yes	No	Can’t Tell	Comments
3.1. Are the participants representative of the target population?	X			
3.2. Are measurements appropriate regarding both the outcome and exposure/intervention?			X	One aspect is unclear, which is the ticket sales data. Ticket sales data seem to be for all routes rather than only the route that received the intervention.
3.3. Are there complete outcome data?			X	Presumably all data from ticket sales are complete. There was no information provided on the response rate or completion rate for the satisfaction surveys.
3.4. Are the confounders accounted for in the design and analysis?		X		
3.5. During the study period, is the intervention/exposure administered as intended?			X	
**Broome et al., 2010** [56] **[Study ID: 1; Paper ID: 1c]**				
Qualitative	Yes	No	Can’t Tell	Comments
1.1. Is the qualitative approach appropriate to answer the research question?	X			
1.2. Are the qualitative data collection methods adequate to address the research question?	X			
1.3. Are the findings adequately derived from the data?	X			
1.4. Is the interpretation of results sufficiently substantiated by data?	X			
1.5. Is there coherence between qualitative data sources, collection, analysis and interpretation?	X			
**Broome et al., 2010** [57] **[Study ID: 1; Paper ID: 1d]**				
Qualitative	Yes	No	Can’t Tell	Comments
1.1. Is the qualitative approach appropriate to answer the research question?	X			
1.2. Are the qualitative data collection methods adequate to address the research question?	X			
1.3. Are the findings adequately derived from the data?	X			
1.4. Is the interpretation of results sufficiently substantiated by data?	X			
1.5. Is there coherence between qualitative data sources, collection, analysis and interpretation?	X			
Quantitative Descriptive	Yes	No	Can’t Tell	Comments
4.1. Is the sampling strategy relevant to address the research question?	X			
4.2. Is the sample representative of the target population?	X			
4.3. Are the measurements appropriate?	X			
4.4. Is the risk of nonresponse bias low?	X			
4.5. Is the statistical analysis appropriate to answer the research question?	X			
Mixed Methods	Yes	No	Can’t Tell	Comments
5.1. Is there an adequate rationale for using a mixed methods design to address the research question?	X			
5.2. Are the different components of the study effectively integrated to answer the research question?	X			
5.3. Are the results adequately brought together into overall interpretations?	X			
5.4. Are divergences and inconsistencies between quantitative and qualitative results adequately addressed?	X			
5.5. Do the different components of the study adhere to the quality criteria of each tradition of the methods involved?	X			
**Nathan et al., 2012** [58] **[Study ID: 2; Paper ID: 2]**				
Quantitative Non-Randomized	Yes	No	Can’t Tell	Comments
3.1. Are the participants representative of the target population?	X			
3.2. Are measurements appropriate regarding both the outcome and exposure/intervention?	X			
3.3. Are there complete outcome data?		X		Missing data were reported.
3.4. Are the confounders accounted for in the design and analysis?		X		Demographic variables were accounted for, but not contextual factors (e.g., community safety, access to other modes of transport; habit of frequenting targeted destinations in other neighbourhoods).
3.5. During the study period, is the intervention/exposure administered as intended?			X	Ideally, the data on household address and neighbourhood destinations are accurate as of the date of data collection for self-reported walking. However, this was not clear.
**Zeitler et al., 2015** [59] **[Study ID: 3; Paper ID: 3a]**				
Qualitative	Yes	No	Can’t Tell	Comments
1.1. Is the qualitative approach appropriate to answer the research question?	X			
1.2. Are the qualitative data collection methods adequate to address the research question?	X			
1.3. Are the findings adequately derived from the data?	X			
1.4. Is the interpretation of results sufficiently substantiated by data?	X			
1.5. Is there coherence between qualitative data sources, collection, analysis and interpretation?	X			
**Zeitler et al., 2012** [60] **[Study ID: 3; Paper ID: 3b]**				
Qualitative	Yes	No	Can’t Tell	Comments
1.1. Is the qualitative approach appropriate to answer the research question?	X			
1.2. Are the qualitative data collection methods adequate to address the research question?	X			
1.3. Are the findings adequately derived from the data?	X			
1.4. Is the interpretation of results sufficiently substantiated by data?	X			
1.5. Is there coherence between qualitative data sources, collection, analysis and interpretation?			X	The comparative aspect is unclear.
**Vine et al., 2012** [61] **[Study ID: 3; Paper ID: 3c]**				
Qualitative	Yes	No	Can’t Tell	Comments
1.1. Is the qualitative approach appropriate to answer the research question?	X			
1.2. Are the qualitative data collection methods adequate to address the research question?	X			
1.3. Are the findings adequately derived from the data?	X			
1.4. Is the interpretation of results sufficiently substantiated by data?	X			
1.5. Is there coherence between qualitative data sources, collection, analysis and interpretation?	X			
**Vine et al., 2012** [62] **[Study ID: 3; Paper ID: 3d]**				
Qualitative	Yes	No	Can’t Tell	Comments
1.1. Is the qualitative approach appropriate to answer the research question?	X			
1.2. Are the qualitative data collection methods adequate to address the research question?	X			
1.3. Are the findings adequately derived from the data?	X			
1.4. Is the interpretation of results sufficiently substantiated by data?	X			
1.5. Is there coherence between qualitative data sources, collection, analysis and interpretation?	X			
**Gardner 2014** [63] **[Study ID: 4; Paper ID: 4]**				
Qualitative	Yes	No	Can’t Tell	Comments
1.1. Is the qualitative approach appropriate to answer the research question?	X			
1.2. Are the qualitative data collection methods adequate to address the research question?	X			
1.3. Are the findings adequately derived from the data?	X			
1.4. Is the interpretation of results sufficiently substantiated by data?	X			
1.5. Is there coherence between qualitative data sources, collection, analysis and interpretation?	X			
**Grant et al., 2010** [64] **[Study ID: 5; Paper ID: 5a**				
Qualitative	Yes	No	Can’t Tell	Comments
1.1. Is the qualitative approach appropriate to answer the research question?	X			
1.2. Are the qualitative data collection methods adequate to address the research question?	X			
1.3. Are the findings adequately derived from the data?	X			
1.4. Is the interpretation of results sufficiently substantiated by data?	X			
1.5. Is there coherence between qualitative data sources, collection, analysis and interpretation?	X			
**Grant et al., 2010** [65] **[Study ID: 5; Paper ID: 5b]**				
Qualitative	Yes	No	Can’t Tell	Comments
1.1. Is the qualitative approach appropriate to answer the research question?	X			
1.2. Are the qualitative data collection methods adequate to address the research question?	X			
1.3. Are the findings adequately derived from the data?	X			
1.4. Is the interpretation of results sufficiently substantiated by data?	X			
1.5. Is there coherence between qualitative data sources, collection, analysis and interpretation?			X	The authors stated that quantitative indicators of the neighbourhood were collected, which implied that they would be analysed. However, it seems that these data were used for context only—to populate descriptive tables. Sample characteristics were also included in descriptive tables yet these were not mentioned in their data collection. In other words, there was inconsistent reporting of methods since the data collected for descriptive tables in one instance were mentioned and not the other.
**Leger et al., 2019** [66] **[Study ID: 6; Paper ID: 6**				
Qualitative	Yes	No	Can’t Tell	Comments
1.1. Is the qualitative approach appropriate to answer the research question?	X			
1.2. Are the qualitative data collection methods adequate to address the research question?	X			
1.3. Are the findings adequately derived from the data?	X			
1.4. Is the interpretation of results sufficiently substantiated by data?	X			
1.5. Is there coherence between qualitative data sources, collection, analysis and interpretation?	X			
**Mah et al., 2017** [67] **[Study ID: 7; Paper ID: 7]**				
Qualitative	Yes	No	Can’t Tell	Comments
1.1. Is the qualitative approach appropriate to answer the research question?	X			
1.2. Are the qualitative data collection methods adequate to address the research question?	X			
1.3. Are the findings adequately derived from the data?	X			
1.4. Is the interpretation of results sufficiently substantiated by data?	X			
1.5. Is there coherence between qualitative data sources, collection, analysis and interpretation?	X			
Quantitative Non-Randomized	Yes	No	Can’t Tell	Comments
3.1. Are the participants representative of the target population?			X	There was no information about the target population nor the selection criteria. While the recruitment methods were described, the authors did not explain what they are recruiting for.
3.2. Are measurements appropriate regarding both the outcome and exposure/intervention?			X	The choice of the measure “You would be impacted if this FTS program was no longer available to you” is not well described. It is unclear what the authors meant by “impact”, which could have been operationalized. It is unclear whether this tool is a standard one or whether it has been validated.
3.3. Are there complete outcome data?			X	
3.4. Are the confounders accounted for in the design and analysis?	X			
3.5. During the study period, is the intervention/exposure administered as intended?			X	
Mixed Methods	Yes	No	Can’t Tell	Comments
5.1. Is there an adequate rationale for using a mixed methods design to address the research question?		X		The authors have not articulated a rationale.
5.2. Are the different components of the study effectively integrated to answer the research question?		X		The two components addressed different aspects of the research question and each had a different sample.
5.3. Are the results adequately brought together into overall interpretations?		X		The two components addressed different aspects of the research question and each had a different sample
5.4. Are divergences and inconsistencies between quantitative and qualitative results adequately addressed?			X	The two components do not overlap.
5.5. Do the different components of the study adhere to the quality criteria of each tradition of the methods involved?		X		See quality appraisal for each component.
**Mitra et al., 2015** [68] **[Study ID: 8; Paper ID: 8]**				
Qualitative	Yes	No	Can’t Tell	Comments
1.1. Is the qualitative approach appropriate to answer the research question?	X			
1.2. Are the qualitative data collection methods adequate to address the research question?	X			
1.3. Are the findings adequately derived from the data?	X			
1.4. Is the interpretation of results sufficiently substantiated by data?	X			
1.5. Is there coherence between qualitative data sources, collection, analysis and interpretation?			X	It is unclear what hand-drawn walking routes and GIS mapping added as the analysis and reporting did not mention these sources. It’s possible that discussion of maps were included in the interviews and therefore transcribed and analysed but this was not clear.
**Ottoni et al., 2017** [69] **[Study ID: 9; Paper ID: 9a]**				
Qualitative	Yes	No	Can’t Tell	Comments
1.1. Is the qualitative approach appropriate to answer the research question?	X			
1.2. Are the qualitative data collection methods adequate to address the research question?	X			
1.3. Are the findings adequately derived from the data?	X			
1.4. Is the interpretation of results sufficiently substantiated by data?	X			
1.5. Is there coherence between qualitative data sources, collection, analysis and interpretation?	X			
**Winters et al., 2015** [70] **[Study ID: 9; Paper ID: 9b]**				
Qualitative	Yes	No	Can’t Tell	Comments
1.1. Is the qualitative approach appropriate to answer the research question?	X			
1.2. Are the qualitative data collection methods adequate to address the research question?	X			
1.3. Are the findings adequately derived from the data?	X			
1.4. Is the interpretation of results sufficiently substantiated by data?	X			
1.5. Is there coherence between qualitative data sources, collection, analysis and interpretation?	X			
Quantitative Descriptive	Yes	No	Can’t Tell	Comments
4.1. Is the sampling strategy relevant to address the research question?			X	The researchers mailed letters to 3402 households with at least one adult aged 60 and over and which was located within ~2 blocks of the planned greenway, but it is not clear how these 3402 households were selected. Recruitment did not identify whether the respondents were cyclist or not. Since cycling behaviour was not asked until the questionnaire, the researchers would not have been in a position to determine if respondents matched the target population and could have ended up in a scenario where all respondents were non-cyclists.
4.2. Is the sample representative of the target population?		X		The target population is described as older adults who live in Vancouver, Canada yet recruitment was targeted at those living in a geographically restricted downtown area next to a planned greenway. Sample descriptive statistics were not compared to that of older adults in Vancouver.
4.3. Are the measurements appropriate?	X			
4.4. Is the risk of nonresponse bias low?		X		Responders and non-responders may differ on cycling behaviours. The information provided in the recruitment letter may have led to selection bias; however, this information was not available.
4.5. Is the statistical analysis appropriate to answer the research question?			X	It is unclear why multivariate analysis was not conducted. There was no information provided as to the type of statistical test conducted.
Mixed Methods	Yes	No	Can’t Tell	Comments
5.1. Is there an adequate rationale for using a mixed methods design to address the research question?		X		The authors have not articulated a rationale.
5.2. Are the different components of the study effectively integrated to answer the research question?		X		The two components addressed different aspects of the research question and each had a different sample.
5.3. Are the results adequately brought together into overall interpretations?		X		The two components addressed different aspects of the research question and each had a different sample
5.4. Are divergences and inconsistencies between quantitative and qualitative results adequately addressed?			X	The two components do not overlap.
5.5. Do the different components of the study adhere to the quality criteria of each tradition of the methods involved?		X		See quality appraisal for each component.
**Adorno et al., 2018** [71] **[Study ID: 10; Paper ID: 10]**				
Qualitative	Yes	No	Can’t Tell	Comments
1.1. Is the qualitative approach appropriate to answer the research question?	X			
1.2. Are the qualitative data collection methods adequate to address the research question?	X			
1.3. Are the findings adequately derived from the data?	X			
1.4. Is the interpretation of results sufficiently substantiated by data?	X			
1.5. Is there coherence between qualitative data sources, collection, analysis and interpretation?	X			
**Gallagher et al., 2010** [72] **[Study ID: 11; Paper ID: 11]**				
Qualitative	Yes	No	Can’t Tell	Comments
1.1. Is the qualitative approach appropriate to answer the research question?	X			
1.2. Are the qualitative data collection methods adequate to address the research question?	X			
1.3. Are the findings adequately derived from the data?	X			
1.4. Is the interpretation of results sufficiently substantiated by data?	X			
1.5. Is there coherence between qualitative data sources, collection, analysis and interpretation?	X			

## Appendix C

The factors that influence older people’s use of each non-motorised transport mode is presented here. Factors are grouped into the categories of the conceptual framework. Factors are presented using a radial dendrogram.

A radial dendrogram is a version of a tree diagram that is useful for depicting hierarchy and classification. The first ring of nodes originating from the center node are categories from the conceptual framework. The second ring of nodes are individual factors. The third ring of nodes are the detailed qualities of individual factors that have a positive (blue) or negative (red) influence. Commonalities with other modes of transportation are also depicted in the outermost layer as text annotations.
